# Phosphorylation of RIPK1 serine 25 mediates IKK dependent control of extrinsic cell death in T cells

**DOI:** 10.3389/fimmu.2022.1067164

**Published:** 2022-12-01

**Authors:** Sam Blanchett, Yves Dondelinger, Alessandro Barbarulo, Mathieu J. M. Bertrand, Benedict Seddon

**Affiliations:** ^1^ Institute of Immunity and Transplantation, The Pears Building, University College London, London, United Kingdom; ^2^ Vlaams Instituut voor Biotechnologie (VIB) Center for Inflammation Research, Ghent, Belgium; ^3^ Department of Biomedical Molecular Biology, Ghent University, Ghent, Belgium

**Keywords:** T cells, extrinsic cell death, RIPK1, IKK complex, TNF

## Abstract

The Inhibitor of Kappa B Kinase (IKK) complex is a critical regulator of NF-κB activation. More recently, IKK has also been shown to repress RIPK1 dependent extrinsic cell death pathways by directly phosphorylating RIPK1 at serine 25. In T cells, IKK expression is essential for normal development in the thymus, by promoting survival of thymocytes independently of NF-κB activation. RIPK1 undergoes extensive phosphorylation following TNF stimulation in T cells, though which targets are required to repress RIPK1 has not been defined. Here, we show that TNF induced phosphorylation of RIPK1 at S25 is IKK dependent. We test the relevance of this phosphorylation event in T cells using mice with a RIPK1^S25D^ phosphomimetic point mutation to endogenous RIPK1. We find that this mutation protects T cells from TNF induced cell death when IKK activity is inhibited *in vitro*, and can rescues development of IKK deficient thymocytes *in vivo* to a degree comparable with kinase dead RIPK1^D138N^. Together, these data show that phosphorylation of RIPK1S25 by IKK represents a key regulatory event promoting survival of T cells by IKK.

## Introduction

The inhibitor of kappa-B kinase (IKK) complex is a trimeric complex of two kinases, IKK1 (IKKα) and IKK2 (IKKβ), and a third regulatory component, NEMO (IKKγ). It is classically described as the prototypic activator of NF-κB transcriptional pathway. IKK phosphorylates inhibitory IκB proteins, targeting them for degradation by the proteasome and thereby releasing NF-κB dimers to enter the nucleus. As such, IKK function has long been presumed to be mediated by its activation of NF-κB family of transcription factors, that play critical roles in controlling development and function of many cell types (1).

In mouse T cells, studies of tissue specific knockouts reveal complex overlapping and distinct roles for IKK and NF-κB in T cell biology. Ablation of the IKK complex, either by deletion of NEMO (2), or combined loss of IKK1 and IKK2 subunits (3) results in a developmental arrest in single positive (SP) thymocytes at the immature HSA^hi^ stage. Similar developmental blocks are observed in mice lacking the upstream activator of IKK, TAK1 (4–6). In contrast, expression of NF-κB REL subunits is redundant for thymic development and generation of mature peripheral T cells (7, 8). An explanation for this apparent contradiction comes from two key observations. First, the trigger for NF-κB activation *via* IKK in developing thymocytes is not TCR but TNF. Blockade of TNF signalling rescues development in IKK1/2 deficient thymocytes (3). Second, recent studies reveal that the IKK complex has two functions during TNF signalling in T cells - activating NF-κB and directly repressing cell death by inhibiting the serine threonine kinase, RIPK1 (8, 9).

Ligation of TNFR1 causes recruitment of TRADD, TRAF2, and the serine/threonine kinase RIPK1. The ubiquitin ligases TRAF2, cellular inhibitor of apoptosis proteins (cIAPs) and the linear ubiquitin chain assembly complex (LUBAC), add ubiquitin chain modifications to themselves and RIPK1, creating a scaffold that allows recruitment and activation of the TAB/TAK and IKK complexes that in turn activate NF-κB. This is termed complex I [reviewed in (10, 11)]. A failure to maintain the stability of this complex results in the formation of cell death inducing complexes. In the presence of IAP inhibitors, IKK inhibitors or TAK1 inhibitors (12–14), a cytosolic protein complex (called complex II) composed of FADD, CASPASE 8 and RIPK1 forms that induces apoptosis, a function dependent upon RIPK1 kinase activity (9, 11, 15). Phosphorylation of RIPK1 by IKK blocks RIPK1 kinase activity and therefore its capacity to induce apoptosis (14, 16). In thymocytes, it is this function of IKK, and not NF-κB activation, that is critical for their survival and accounts for the phenotype observed in IKK deficiency (8).

Since the recognition that RIPK1 activity can be directly controlled through phosphorylation, there has been considerable interest in better understanding those kinases that target RIPK1 and the impact on RIPK1 function. RIPK1 appears to undergo extensive phosphorylation on multiple residues during TNF signalling (17) that have been implicated in repressing the kinase activity of RIPK1. In addition to autophosphorylation (18), TAK1, TBK1, MK2 and IKK have all been shown in various cell types and contexts to mediate repressive phosphorylation of RIPK1 (14, 19–24). IKK has been shown to phosphorylate RIPK1 both at Ser6 and Ser25 (14), and it is this latter site that has been shown to be important to prevent cell death in MEFs and during *Yersinia* infection (16), in which effective immune control of infection depends on RIPK1 triggered cell death processes in myeloid cells (25). In T cells, the minimal requirements for repression of RIPK1 have not been defined. In the present study, we probed the mechanism by which IKK represses RIPK1 specifically in T cells by asking whether phosphorylation of Ser25 of RIPK1 was required and sufficient for the pro-survival functions of IKK function in T cells, both during TNF stimulation *in vitro*, and in mice with IKK deficient T cells *in vivo*.

## Results

### IKK function and expression is essential for phosphorylation of RIPK1Ser25

**Figure 1:**
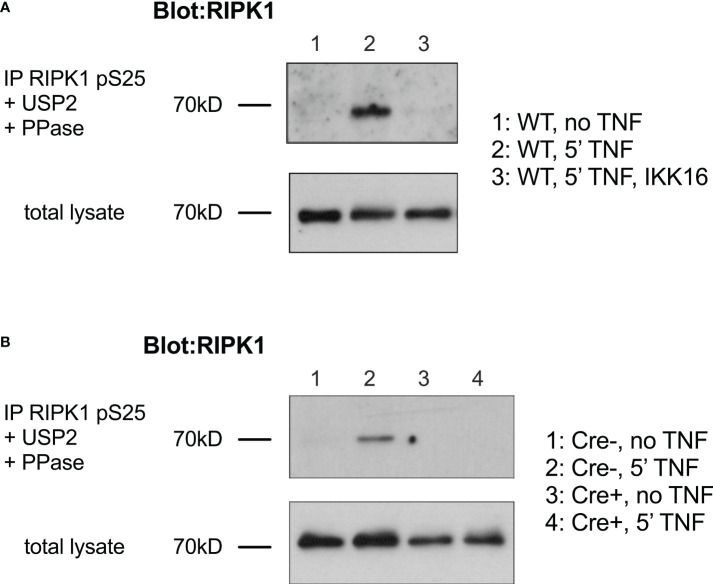
Phosphorylation of RIPK1 Ser25 following TNF stimulation is IKK dependent in T cells. **(A)** Thymocytes from WT mice were stimulated for 5’ with PBS (lane 1) or TNF (lane 2-3) (5ng/ml) following pre-incubation for 30’ with IKK16 (2µM, lane 3). Cell were lysed and immunoprecipitated with anti-phospho-Ser25 RIPK1 (RIPK1 pS25), followed by treatment with USP2 and PPase. i.p. were separated by gel electrophoresis and RIPK1 protein analysed by western blot. Corresponding total cell lysates were run prior to immunoprecipitation as loading control. **(B)** Thymocytes from Cre+ and Cre- littermate IKK1/2^ΔCD2^ RIPK1D138N mice were stimulated with TNF and immunopreciptates of anti-RIPK1 pS25 analysed by immunoblotting as described in **(A)**. Data are representative of one other experiment.

Our previous work shows that following TNF stimulation in T cells, phosphorylation of RIPK1 within complex I is dependent upon IKK (8). We therefore asked whether Ser 25 of RIPK1 was phosphorylated in mouse T cells and if it was dependent on IKK activity. Mouse thymocytes were stimulated with TNF and then cytosolic extracts analysed by immunoblotting. Shortly after TNF stimulation and mouse thymocytes, RIPK1 is recruited to TNFR1 complex I and becomes modified with extensive ubiquitin chain additions and phosphorylation residues. To detect pS25 RIPK1, a phospho-specific anti-RIPK1 pSer25 anti-sera was used to immunoprecipitate pS25 RIPK1 from cell extracts (16). Immunoprecipitates were subsequently treated to remove ubiquitin and phospho groups and then total RIPK1 detected by immunoblotting. Following TNF stimulation of WT thymocytes, pS25 RIPK1 was readily detectable (**Figure 1A**). Pretreatment of cells with pan-IKK inhibitor, IKK16, prior to TNF stimulation, prevented detection of pS25 RIPK1, demonstrating that pS25 was IKK dependent. To confirm the requirement for IKK genetically, we wished to assess TNF stimulation of thymocytes lacking IKK expression. However, since IKK deletion results in a complete loss of mature thymocytes, we analysed *Chuk^fx/fx^ Ikbkb^fx/fx^* huCD2^iCre^ (IKK1/2^ΔCD2^) mice that express a kinase dead RIPK1^D138N^ mutant. Inactivation of RIPK1 *in vivo* in this way in IKK1/2^ΔCD2^ RIPK1^D138N^ mice rescues thymocytes from cell death, and permits analysis of RIPK1 recruitment to TNF induced complex I (8). Following TNF stimulation, pS25 RIPK1 was readily detectable in thymocytes from Cre -ve litter mate controls. In contrast, no pS25 RIPK1 was detected following TNF stimulation of IKK1/2 deficient thymocytes (**Figure 1B**). Together, these data demonstrate that Ser25 of RIPK1 is a target of phosphorylation following TNF stimulation of thymocytes, and that phosphorylation is strictly IKK dependent.

### TNF induced cell death of thymocytes is completely blocked in RIPK1^S25D^ expressing mice

We next wanted to ask whether IKK dependent phospshorylation of RIPK1Ser25 was sufficient for repression of TNF induced cell death by IKK. To do this, we took advantage of a recently described knockin mutant mouse strain that expresses an RIPK1^S25D^ mutant (16). In this strain, Ser25 has been mutated to aspartic acid. Aspartic acid and phosphorylated serine closely resemble one another chemically, and this mutation has been shown to have the predicted phosphomimetic properties (16). We therefore asked whether this phosphomimetic of RIPK1 Ser25 was sufficient to prevent RIPK1 induced cell death in the absence of IKK activity in T cells. Since IKK1/2^ΔCD2^ mice lack mature thymocytes and have no peripheral T cells, we took a combined genetic and pharmacological approach to test the efficacy of RIPK1^S25D^ in blocking cell death. We generated IKK1^ΔCD2^ RIPK1^S25D^ that specifically lack IKK1 but express IKK2. Redundancy between IKK1 and IKK2 permits near normal development and generation of T cells in the absence of IKK1 expression (3, 8, 26). Application of the highly specific IKK2 inhibitor, Bl605906, (27) to IKK1 deficient T cells results in a robust blockade of IKK activity.

**Figure 2:**
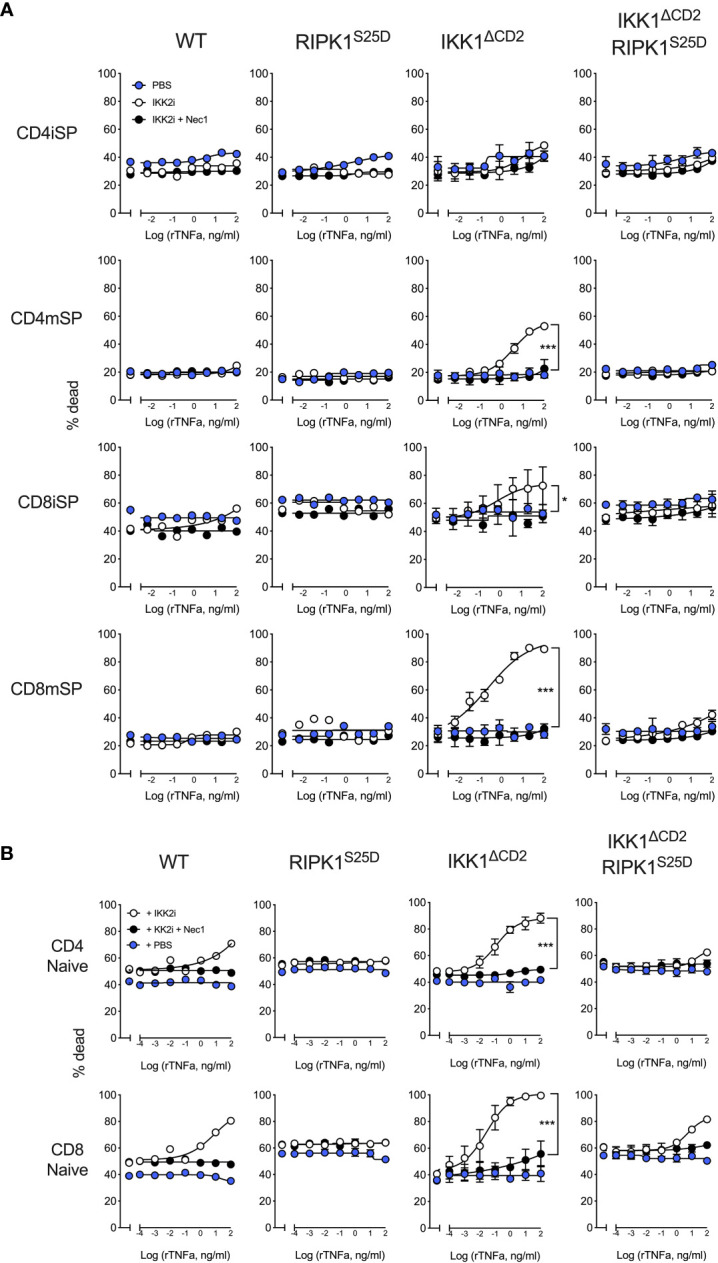
TNF induced cell death in the absence of IKK kinase activity is blocked by RIPK1^S25D^ in thymocytes and peripheral naive T cells. Thymus and LN cells from IKK1^ΔCD2^ and IKK1^ΔCD2^RIPK1^S25D^ mice, and iCre -ve litter mates (WT or RIPK1^S25D^ columns), were cultured for 24h at different doses of TNF with addition of either IKK2i (10µM), IKK2i + Nec1 (10nM), or PBS as control. Cultured cells were analysed by flow cytometry and % dead cells calculated and graphed for the indicated populations of thymocytes **(A)** and peripheral naive T cells **(B)**. Data are representative of three or more independent replicate experiments. Error bars show s.d. Significant differences between PBS and IKK2i conditions were determined by 2-way ANOVA. * < 0.05, *** < 0.001.

We first tested the capacity of RIPK1^S25D^ to inhibit TNF induced apoptosis in thymocytes *in vitro*. Our previous work shows that thymocytes become progressively more dependent upon IKK signalling to promote survival as they transition through development, correlating with induction of RIPK1 protein expression (8). CD4^+^CD8^+^ double positive (DP) thymocytes and immature HSA^hi^ CD4^+^ single positive (CD4iSP) thymocytes do not require IKK for survival *in vivo*. In contrast, immature HSA^hi^ CD8^+^ SP (CD8iSP) thymocytes and both mature HSA^lo^ CD4^+^ (CD4mSP) and HSA^lo^ CD8^+^ SP (CD8mSP) thymocytes are all highly dependent on IKK expression for their survival *in vivo* (3). To assess TNF induced death, thymocytes from WT, IKK1^ΔCD2^ and IKK1^ΔCD2^RIPK1^S25D^ mice were cultured overnight with different doses of TNF in the presence and absence of IKK2 inhibitor and cell death assessed by flow cytometry. IKK2 inhibition alone did not render any subset of thymocytes susceptible to TNF induced cell death (**Figure 2A**). In contrast, in the presence of IKK2 inhibitor, CD8iSP, CD8mSP and CD4mSP underwent increasing extents of cell death with increasing doses of TNF. Cell death was RIPK1 kinase dependent, since further application of RIPK1 inhibitor, Nec1, completely blocked cell death in cultures. In contrast, no TNF inducible cell death was observed in cultures of thymocytes from IKK1^ΔCD2^ RIPK1^S25D^ mice with IKK2 inhibitor. We presume that cell death was mediated by apoptosis rather than necroptosis, since thymocytes do not express MLKL (8), and previous studies show that T cells become susceptible to necroptosis only following activation (28).

### TNF induced cell death of peripheral T cells is largely blocked by RIPK1^S25D^

We next tested whether RIPK1^S25D^ was sufficient to also protect mature peripheral T cells from TNF induced cell death. In contrast to thymocytes, IKK2 inhibitor alone was able to render WT T cells susceptible to RIPK1 dependent TNF induced cell death at higher concentrations of TNF. In contrast, T cells from RIPK1^S25D^ mice expressing normal IKK, were resistant to cell death in the face of IKK2 inhibition (**Figure 2B**). Similarly, IKK2 inhibition of IKK1 deficient peripheral T cells from IKK1^ΔCD2^ mice resulted in high levels of cell death, even at lower concentrations of TNF. In this IKK1 deficient setting, RIPK1^S25D^ rescued both CD4^+^ and CD8^+^ T cells from TNF induced cell death at all but the highest concentrations of TNF, where reproducible low levels of cell death were observed. To test whether the low level of cell death observed in cultures of T cells from IKK1^ΔCD2^RIPK1^S25D^ mice with IKK2 inhibitor were RIPK1 dependent, we also cultured cells in the presence of Nec1. This showed that the low level of cell death of TNF stimulated IKK1^ΔCD2^RIPK1^S25D^ was in fact RIPK1 dependent, suggesting some residual kinase activity of RIPK1^S25D^.

### RIPK1^S25D^ rescues development of IKK deficient thymocytes

**Figure 3:**
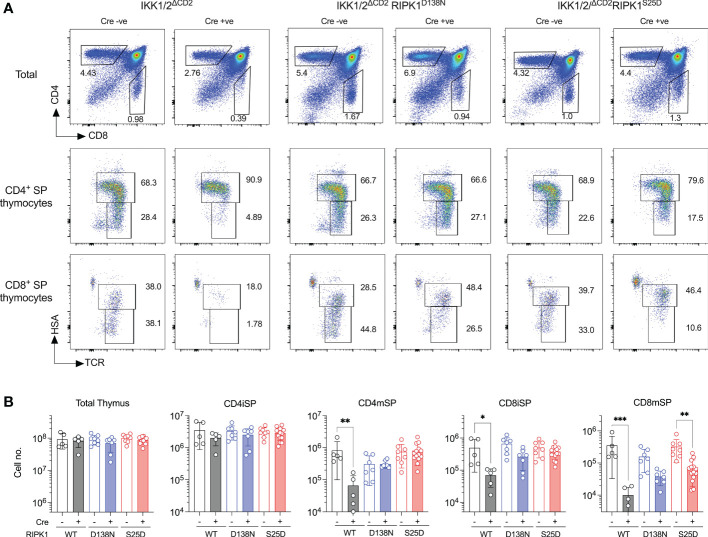
RIPK1^S25D^ rescues IKK deficient thymocytes from cell death *in vivo*. Single cell suspensions were prepared from the thymus of Cre -ve (n=5) and Cre +ve (n=5) IKK1/2^ΔCD2^, Cre -ve (n=7) and Cre +ve (n=7) IKK1/2^ΔCD2^ RIPK1^D138N^ and Cre -ve (n=8) and Cre +ve (n=14) IKK1/2^ΔCD2^ RIPK1^S25D^ mice, enumerated and analyse by flow cytometry. **(A)** Flow plots show phenotype of the indicated populations (rows) from the indicated mice (columns) and gates used to define immature HSA^hi^ CD4^+^ TCR^hi^ SP (CD4iSP), mature HSA^lo^ CD4^+^ TCR^hi^ SP (CD4mSP) immature HSA^hi^ CD8^+^ TCR^hi^ SP (CD8iSP) and HSA^lo^ CD8^+^ TCR^hi^ SP (CD8mSP) thymic subpopulations. **(B)** Bar charts show the total cell numbers for the indicated populations recovered from different strains. Data are pooled from five independent experiments. Mann-Witney student’s t test. *p < 0.05, **p < 0.01, ***p < 0.001.

To test the capacity of RIPK1^S25D^ to block cell death *in vivo*, we asked whether the block in T cell development in IKK1/2^ΔCD2^ mice could be overcome by the phosphomimetic RIPK1 mutant. We therefore generated IKK1/2^ΔCD2^RIPK1^S25D^ and compared their phenotype with IKK1/2^ΔCD2^ mice expressing a kinase dead RIPK1^D138N^ mutant. As previously reported, numbers of DP and CD4iSP thymocytes are normal in the absence of IKK expression, while CD8iSP, CD8mSP and CD4mSP subsets are profoundly reduced in numbers (**Figure 3**). Kinase dead RIPK1 rescues numbers of CD4mSP and CD8iSP to near normal levels, while CD8mSP numbers are restored to levels approximately half way between those in IKK1/2^ΔCD2^ and WT (8). Analysing thymus of IKK1/2^ΔCD2^RIPK1^S25D^ mice revealed an identical pattern of rescue to the kinase dead RIPK1 expressing mice. CD4mSP and CD8iSP numbers were indistinguishable from IKK expressing controls, while CD8mSPs underwent a clear but incomplete rescue of cellularity (**Figure 3**). These results suggest that RIPK1^S25D^ mutant is sufficient to repress RIPK1 dependent cell death *in vivo* in the absence of IKK expression.

### RIPK1^S25D^ rescues CD4^+^ but not CD8^+^ peripheral T cells in IKK1/2^ΔCD2^ mice

**Figure 4:**
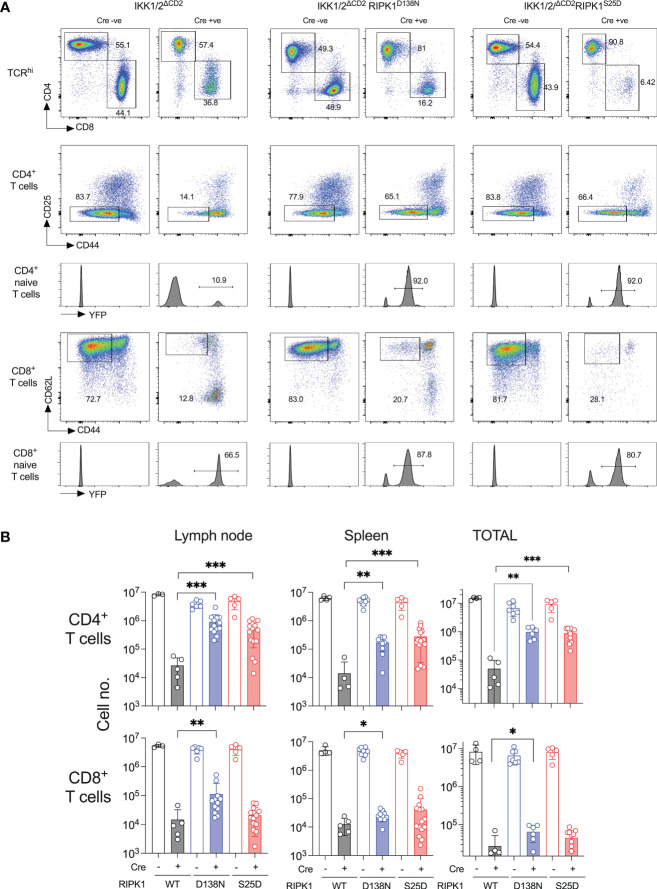
RIPK1^S25D^ rescues IKK deficient CD4^+^ but not CD8^+^ naive T cells from cell death *in vivo*. LN and spleen were recovered from the same groups of mice described in **Figure 3** and single cell suspensions analysed by flow cytometry. **(A)** Flow plots show the phenotype of lymph node cells and gates used to identify CD4^+^ naive T cells as CD4^+^ TCR^hi^ CD44^lo^ CD25^-^ and CD8^+^ naive T cells as CD8^+^ TCR^hi^ CD44^lo^ CD25^-^ cells. Histograms are of YFP expression by naive gated T cells from the corresponding strains. YFP is only activated in Cre expressing strains. **(B)** Bar charts show the numbers of CD4^+^ and CD8^+^ naive T cells recovered from lymph nodes, spleen and both combined, from the indicated strains. Cell recoveries from Cre+ and corresponding Cre- strains were all highly significant differences (p<0.0001). Significant differences between Cre+ strains are indicated on plots. Data are pooled from five independent experiments. * p<0.05, ** p<0.01, *** p<0.001.

Earlier work shows that kinase dead RIPK1 permits a substantial rescue of CD4^+^ naive T cell compartment and a small but significant rescue of CD8^+^ naive T cells (8). We therefore compared the size and composition of the peripheral T cell compartments of IKK1/2^ΔCD2^RIPK1^D138N^ and IKK1/2^ΔCD2^RIPK1^S25D^ mice. Substantial naive CD4^+^ compartments were evident in both IKK1/2^ΔCD2^RIPK1^D138N^ and IKK1/2^ΔCD2^RIPK1^S25D^ mice (**Figure 4A**), with similar numbers of recovered in both strains (**Figure 4B**). Significant rescue of CD8^+^ naive T cells was evident, albeit at a lower level than observed in the CD4^+^ compartment, in IKK1/2^ΔCD2^RIPK1^D138N^ mice. There was some evidence of rescue in CD8^+^ naive T cell numbers in IKK1/2^ΔCD2^RIPK1^S25D^ mice, but not to significant levels. The various IKK1/2^ΔCD2^ strains also carried a Rosa26^RYFP^ Cre reporter construct. This is useful in the context of gene deletions resulting in strong developmental blocks, because rare cells in which iCre fails to completely delete conditional genes, so called ‘escapants’, can have a competitive advantage over gene deleted counterparts, and may accumulate in peripheral lymphoid tissues. Indeed, there are substantial fractions of YFP-ve cells amongst the naive T cells present in periphery of IKK1/2^ΔCD2^ mice, indicating the presence of such escapants. Analysing YFP expression by naive T cells in IKK1/2^ΔCD2^RIPK1^D138N^ and IKK1/2^ΔCD2^RIPK1^S25D^ mice revealed a strong restoration of YFP expressing cells, further indicating a degree of rescue of gene deleted populations in these mice. In contrast, amongst effector phenotype and CD25^+^ Treg populations, YFP expression remained less than 5% in all IKK deficient strains (data not shown) indicating no rescue of these subsets by either RIPK1^S25D^ or RIPK1^D138N^ mutants. Nevertheless, rescue of CD4^+^ naive T cells in both RIPK1 mutant strains was far more extensive than observed in CD8^+^ subsets.

## Discussion

Here, we investigated the mechanisms by which IKK signalling regulates RIPK1 dependent cell death in T cells, specifically whether phosphorylation of Ser25 of RIPK1 by IKK was sufficient to mediate repression of RIPK1. We make the following novel findings. We show that RIPK1 Ser25 is a target of phosphorylation in T cells following TNF stimulation, and that phosphorylation is dependent upon both IKK expression and kinase activity. Analysing mice expressing a phosphomimetic S25D mutant of RIPK1, suggests that phosphorylation of Ser25 is sufficient to almost completely block TNF induced cell in the absence of IKK activity *in vitro* and show that the same mutant mediates a near identical rescue of thymocyte development in IKK deficient T cells as kinase dead RIPK1^D138N^ mutant. Together, these results highlight Ser25 of RIPK1 as a critical regulatory target of IKK kinase activity in T cells.

In thymocytes, RIPK1^S25D^ was sufficient to completely block TNF induced cell death *in vitro*. In contrast, peripheral T cells from RIPK1^S25D^ mice, under conditions of IKK inhibition, underwent a low level of TNF induced cell death that was apparently RIPK1 kinase dependent, since death was blocked by Nec1. This highlights some features of IKK regulated cell death in T cells noted elsewhere (29), specifically that repression of cell death by the IKK complex in peripheral T cells has more stringent requirements for IKK activity than in thymocytes. There is redundancy between IKK1 and IKK2 to control RIPK1 in thymocytes. The activity of IKK1 alone appears sufficient to control RIPK1 mediated cell death in thymocytes, since IKK2 inhibitor did not sensitise any population to cell death, and similarly, IKK1 deficient thymocytes were resistant to TNF induced cell death. In contrast, IKK2 inhibition did sensitise WT peripheral T cells, and to a greater extent in CD8^+^ T cells than in CD4^+^ T cells. Therefore, the incomplete inhibition of cell death by RIPK1^S25D^ amongst CD8^+^ T cells may reflect more active engagement of extrinsic cell death pathways specifically in CD8^+^ T cells. *Ripk1* expression is induced during thymic development and reaches a greater maximal level in CD8 lineage cells than CD4. Higher intracellular concentrations of RIPK1 may increase the likelihood of triggering cell death pathways given appropriate stimuli, and may require a higher threshold IKK kinase activity to maintain effective repression. This would explain why active repression of TNF induced cell death is more easily perturbed by IKK2 inhibitor in CD8 peripheral T cells.

The impact of RIPK1^S25D^ mutant upon TNF induced cell death we observed *in vitro*, strongly correlated with the capacity of this mutant to rescue thymocytes and T cells from the consequences of IKK ablation *in vivo*. CD4mSP thymocytes and peripheral naive CD4^+^ T cells were equally well rescued by RIPK1^S25D^ and RIPK1^D138N^. In contrast, the failure of RIPK1^S25D^ to completely block TNF induced cell death following IKK inhibition in CD8^+^ T cells *in vitro* was reflected in the *in vivo* phenotype of IKK1/2^ΔCD2^RIPK1^S25D^ mice. While RIPK1^S25D^ and RIPK1^D138N^ mediated comparable rescue of CD8^+^ thymic subsets, we did not observed any significant rescue of CD8^+^ naive T cells by RIPK1^S25D^, although enrichment of YFP expressing cells in this compartment was suggestive of some low level of rescue. These observations suggest that the modest level of TNF induced cell death observed *in vitro* in RIPK1^S25D^ mice was physiologically relevant, under conditions of complete IKK ablation *in vivo*, and highlights that reactivity of T cells to TNF stimulation *in vitro* can be accurately predictive *in vivo* behaviour.

Blockade of TNF induced cell death in RIPK1^S25D^ cells by Nec1, revealed that kinase activity of the RIPK1^S25D^ mutant is not completely repressed by this mutation. Ser25 of RIPK1 is within the kinase domain of RIPK1, next to highly conserved Glycine residues thought to facilitate the γ- phosphate of ATP for catalysis. The phosphorylation of Ser25 is thought to sterically interfere with ATP substrate access to the enzymatic domain of RIPK1. It remains unclear whether the S25D RIPK1 mutation represents a perfect phosphomimetic, and that RIPK1 with phospho-Ser25 alone retains some level of kinase activity or whether RIPK1 induced cell death simply represents imperfect repression of kinase activity by S25D mutation. Other studies show that Ser6, that is also phosphorylated by IKK, does not contribute to repression of RIPK1, either alone or in combination with Ser25 (16). However, while RIPK1^S25D^ alone mediates very substantial protection of T cells from the impact of IKK blockade, and suggests that survival of T cells requires tonic phosphophorylation of RIPK1 on this residue, it is possible that optimal repression also requires inhibitory modifications by other kinases to other residues. It will be important in future studies to determine the minimal modifications required for repression of RIPK1, as well as the minimal deregulation required to unleash RIPK1 induced cell death. For instance, TAK1 is required to activate IKK complex but has also been implicated in other cell types of directly phosphorylating RIPK1 and mediating repression. TAK1 deletion in T cells results in a related, yet distinct, arguably more profound thymic block than observed following IKK deletion (6). While a failure to activate IKK undoubtedly contributes to the phenotype of TAK1 deletion, the capacity of TAK1 to activate other pathways, such as p38-MK2 that results in phosphorylation of RIPK1 (19, 21), may create a compound phenotype, that may include targeting RIPK1, both indirectly through IKK and MK2 activation, and also by direct kinase activity. It is unclear whether optimal RIPK1 repression in T cells requires cooperative activity of both IKK and TAK1, or whether these kinases activities mediate repression independently of one another. Disentangling such regulatory networks controlling RIPK1 activity remain important future challenges.

## Materials and methods

### Mice

Mice with conditional alleles of *Ikbkb* (30) and/or *Chuk* (31) were intercrossed with mice expressing *Cre* under the control of the human CD2 (*huCD2^iCre^*) (32), and with mice with a D138N mutation in *Ripk1* (RIPK1^D138N^) (33) or with a *Ripk1^S25D^* mutation (16). *Chuk^fx/fx^ huCD2^iCre^* (IKK1^ΔCD2^), *Chuk^fx/fx^ Ikbkb^fx/fx^ huCD2^iCre^* (IKK1/2^ΔCD2^), *Chuk^fx/fx^ Ikbkb^fx/fx^ huCD2^iCre^* RIPK1^D138N^ (IKK1/2^ΔCD2^ RIPK1^D138N^), *Chuk^fx/fx^ Ikbkb^fx/fx^ huCD2^iCre^* RIPK1^S25D^ (IKK1/2^ΔCD2^ RIPK1^D138N^), *Chuk^fx/fx^ huCD2^iCre^* RIPK1^S25D^(IKK1^ΔCD2^ RIPK1^S25D^) strains were bred in the Comparative Biology Unit of the Royal Free UCL campus and at Charles River laboratories, Manston, UK. Animal experiments were performed according to institutional guidelines and Home Office regulations.

### Flow cytometry and electronic gating strategies

Flow cytometric analysis was performed with 2-5 x 10^6^ thymocytes, 1-5 x 10^6^ lymph node or spleen cells. Cell concentrations of thymocytes, lymph nodes (superficial cervical, mandibular, axillary, superficial inguinal, and mesenteric chain) and spleen cells were determined with a Scharf Instruments Casy Counter. Cells were incubated with saturating concentrations of antibodies in 100 μl of Dulbecco’s phosphate-buffered saline (PBS) containing bovine serum albumin (BSA, 0.1%) for 1hour at 4°C followed by two washes in PBS-BSA. Panels used the following mAb: EF450-conjugated antibody against CD25(ThermoFisher Scientific), PE-conjugated antibody against CD127 (ThermoFisher Scientific), BV785-conjugated CD44 antibody (Biolegend), BV650-conjugated antibody against CD4 (Biolegend), BUV395-conjugated antibody against CD8 (BD Biosciences), BUV737-conjugated antibody against CD24 (BD Biosciences), PerCP-cy5.5-conjugated antibody against TCR (Tonbo Biosciences). Cell viability was determined using LIVE/DEAD cell stain kit (Invitrogen Molecular Probes), following the manufacturer’s protocol. multi-color flow cytometric staining was analyzed on a LSRFortessa (Becton Dickinson) instrument, and data analysis and color compensations were performed with FlowJo V10 software (TreeStar). Naive peripheral T cells were identified by gating CD4^+^ or CD8^+^ subsets with TCR^hi^ CD44^lo^ CD25^lo^. Mature CD4^+^ and CD8^+^ SP thymocytes were identified as TCR^hi^CD4^+^CD8^-^HSA^lo^ and TCR^hi^CD4^-^CD8^+^HSA^lo^ respectively.

### *In vitro* culture

Thymocytes and LN T cells were cultured at 37°C with 5% CO2 in RPMI-1640 (Gibco, Invitrogen Corporation, CA) supplemented with 10% (v/v) fetal bovine serum (FBS) (Gibco Invitrogen), 0.1% (v/v) 2-mercaptoethanol βME (Sigma Aldrich) and 1% (v/v) penicillin-streptomycin (Gibco Invitrogen). Recombinant TNF (Peprotech) was supplemented to cultures at 20ng/ml, unless otherwise stated, with PBS used as vehicle. Inhibitors were used at the following concentrations, unless otherwise stated: IKK2 inhibitor BI605906 (Tocris Bio-techne) (IKK2i) (10µM in 0.1% DMSO vehicle), Nec1 (10µM in 0.1% DMSO) (Insight biotechnology).

### Immunoblotting

Thymocytes (2 x 10^7^/condition) were stimulated with TNF (50ng/ml) for 5 minutes, washed two times in ice-cold PBS and lysed in NP-40 lysis buffer. pSer25 RIPK1 complexes were immunoprecipitated, followed by deubiquitylated (by USP2 treatment) and dephosphorylated (by lambda protein phosphatase treatment) as described elsewhere (16). Enzymatic reactions were allowed to proceed for 30 min at 37°C and subsequently quenched by the addition of 12.5 µL 5x laemmli buffer. IPs and total cell lysates were analyzed by standard immunoblotting. Generation of anti-pSer25 RIPK1 sera and its utility for detecting pSer25 RIPK1 by immunoprecipitation is characterised and described in detail elsewhere (16).

### Statistics

Statistical analysis, line fitting, regression analysis, and figure preparation were performed using Graphpad Prism 8. Column data compared by unpaired Mann-Witney student’s t test. * p<0.05, ** p<0.01, *** p<0.001, **** p < 0.0001.

## Data availability statement

The raw data supporting the conclusions of this article will be made available by the authors, without undue reservation.

## Ethics statement

The animal study was reviewed and approved by United Kingdom Home Office under project license PPL PP2330953.

## Author contributions

Conceptualization: MB and BS; Methodology: SB, YD, AB, MJMB, and BS; Investigation: SB, YD, and AB; Visualization: SB, YD, and BS; Funding acquisition: BS; Project administration: SB and BS; Supervision: BS; Writing – original draft: BS; Writing – review and editing: BS, SB, YD, and MB. All authors contributed to the article and approved the submitted version.

## Funding

The work in the Seddon lab is supported by the Medical Research Council UK under programme codes MR/P011225/1 and MR/N013867/1. Research in the lab of MJMB is supported by the VIB, by Ghent University (iBOF ATLANTIS), by grants from the FWO (G035320N, G044518N, EOS G0G6618N, EOS G0I5722N) and from the Flemish Government (Methusalem BOF16/MET_V/007 - attributed to P. Vandenabeele).

## Acknowledgments

We thank UCL Comparative Biology Unit staff for assistance with mouse breeding and maintenance. We thank the following for generously sharing of their mouse strains: Prof Manolis Pasparakis for *Chuk* conditional strain, Prof Michael Karin for *Ikbkb* conditional strain, Prof Vishva Dixit for the RIPK1^D138N^ strain.

## Conflict of interest

The authors declare that the research was conducted in the absence of any commercial or financial relationships that could be construed as a potential conflict of interest.

## Publisher’s note

All claims expressed in this article are solely those of the authors and do not necessarily represent those of their affiliated organizations, or those of the publisher, the editors and the reviewers. Any product that may be evaluated in this article, or claim that may be made by its manufacturer, is not guaranteed or endorsed by the publisher.
